# Intracameral moxifloxacin for endophthalmitis prophylaxis after cataract surgery: a systematic review and meta-analysis

**DOI:** 10.3389/fmed.2025.1704056

**Published:** 2026-01-08

**Authors:** Ahmed Abu-Zaid, Abdullah M. H. E. Alkandari, Zainab A. R. Hubail, Mshari S. Alenezi, Deemah Alkandari, Rashed A. Alasoosi, Danah Jamil Hammadi, Abdulwahab Q. Majeed, Abdulwahab Alqallaf, Fatmah S. Semairan, Abdulwahab Alhumaidi, Abdullah M. Alharran

**Affiliations:** 1 Department of Biochemistry and Molecular Medicine, College of Medicine, Alfaisal University, Riyadh, Saudi Arabia; 2 Faculty of Medicine, University of Jordan, Amman, Jordan; 3 Faculty of Medicine, Mansoura University, Mansoura, Egypt; 4 Ministry of Health, Kuwait City, Kuwait; 5 College of Medicine and Medical Sciences, Arabian Gulf University, Manama, Bahrain; 6 Royal College of Surgeons in Ireland, Manama, Bahrain

**Keywords:** antibiotic, infection, inflammation, lens, phacoemulsification

## Abstract

**Background:**

Postoperative endophthalmitis is a devastating complication of cataract surgery. Intracameral moxifloxacin has emerged as a promising prophylactic strategy due to its broad-spectrum properties and pre-formulated preparations. However, a robust synthesis of evidence from randomized controlled trials (RCTs) is needed to confirm its efficacy and safety.

**Methods:**

A systematic review and meta-analysis were conducted using evidence from PubMed, Scopus, Web of Science, and CENTRAL, including RCTs published up to August 2025. The primary outcome was the incidence of endophthalmitis, while secondary outcomes included endothelial cell count (ECC) and central corneal thickness (CCT). We pooled outcomes using risk ratios (RRs) and mean differences (MDs) with 95% confidence intervals (CIs) using Stata (version 18).

**Results:**

A total of six RCTs involving 4,438 patients were included. Overall, one RCT demonstrated a low risk of bias, three RCTs raised some concerns, and two RCTs were assessed as having a high risk of bias. Intracameral moxifloxacin significantly reduced the incidence of postoperative endophthalmitis compared to the control group (*n* = 5 RCTs, RR: 0.22, 95% CI [0.07, 0.77], *p* = 0.02). A sensitivity analysis excluding studies with a high risk of bias demonstrated that the effect remained statistically significant (*n* = 3 RCTs, RR: 0.183, 95% CI 0.038, 0.874, *p* = 0.03), with no evidence of heterogeneity (*I*^2^ = 0%, *p* = 0.65). There was no significant difference between the moxifloxacin and control groups regarding postoperative changes in ECC (*n* = 3 RCTs, MD: 22.17, 95% CI [−8.53, 52.88], *p* = 0.16) or CCT (*n* = 3 RCTs, MD: -0.03, 95% CI [−0.36, 0.31], *p* = 0.88).

**Conclusion:**

Prophylactic intracameral moxifloxacin significantly reduces the incidence of postoperative endophthalmitis following cataract surgery. This substantial protective benefit is achieved without evidence of compromised endothelial safety; however, safety conclusions are limited by the small number of patients assessed and should be interpreted with caution.

**Systematic review registration:**

The study was registered in the International Prospective Register of Systematic Reviews (PROSPERO) (ID: CRD420251144067), https://www.crd.york.ac.uk/PROSPERO/view/CRD420251144067.

## Introduction

Cataract surgery is one of the most commonly performed surgical procedures globally, with over 20 million annual operations and a high success rate (1). Postoperative endophthalmitis remains a rare yet devastating complication, despite significant advances in surgical techniques and safety measures (2). This acute intraocular inflammation may result in permanent vision loss, making its prevention a serious concern for ophthalmologists. Infection stems principally from the patient’s ocular surface flora, which gains access to the eye during or after the operation (3). Therefore, appropriate antimicrobial prophylaxis is considered a necessary aspect of the surgical standard of care, even when infection rates are low (4).

Over the past few decades, standards of care have changed. Preoperative application of povidone–iodine to the ocular surface is an evidence-based measure with universal acceptance (5). However, a notable shift in practice occurred with the introduction of intracameral antibiotics, supported by the European Society of Cataract and Refractive Surgeons (ESCRS) multi-center study (6), which reported that intracameral cefuroxime reduced the risk of postoperative endophthalmitis by nearly fivefold (6). Despite this compelling evidence, several practical challenges have prevented the adoption of intracameral cefuroxime. As it is not commercially available as a single-use, pre-formulated intraocular solution in many parts of the world, cefuroxime requires manual reconstitution from powder, which introduces the risks of dilution errors, incorrect dosing, and microbial contamination (7). In addition, cefuroxime has recognized limitations in its antimicrobial spectrum, demonstrating insufficient efficacy against notable ocular pathogens, including *Enterococcus* species and *Pseudomonas aeruginosa* (8). Furthermore, the lack of an approved, readily available formulation in major regions, such as the United States, remains a significant barrier to its regular application (9).

Due to these limitations, the search for a better alternative identified moxifloxacin as an attractive and promising option (10). As a fourth-generation fluoroquinolone, moxifloxacin exhibits comprehensive bactericidal activity against many Gram-positive and Gram-negative bacteria implicated in postoperative endophthalmitis. Another significant advantage of its intracameral use is its commercial formulation (e.g., Vigamox, Alcon). It is a sterile, self-preserved, isotonic ophthalmic solution with a pH similar to that of the eye, eliminating the need for manual preparation and dilution, which can be logistically challenging and pose safety issues (11). Prophylactic intracameral moxifloxacin appears to be safe and highly effective in decreasing the incidence of endophthalmitis, according to an expanding body of evidence, particularly from large retrospective cohort studies (12, 13).

Despite promising retrospective data, a definitive conclusion regarding the efficacy and safety of intracameral moxifloxacin requires a rigorous synthesis of the highest-quality clinical evidence, namely randomized controlled trials (RCTs) (11, 14–18). Therefore, we conducted this systematic review and meta-analysis of available RCTs to comprehensively evaluate the efficacy and safety of prophylactic intracameral moxifloxacin in patients undergoing cataract surgery.

## Methods

### Protocol registration

We registered this systematic review in the International Prospective Register of Systematic Reviews (PROSPERO) under the registration number CRD420251144067. The methodology for this systematic review and meta-analysis adhered to the PRISMA guidelines (19) and the Cochrane Handbook for Systematic Reviews of Interventions (20).

### Data source and search strategy

On 20 August 2025, a systematic literature search was conducted across the following electronic databases: PubMed, Scopus, Web of Science, CENTRAL, and Google Scholar. The search strategy utilized the following keywords: “(Moxifloxacin OR Avelox OR Moxeza OR Vigamox OR Actira OR Izilox OR Octegra OR Proflox) AND (“Cataract Extract*” OR Phacoemulsification OR “Cataract Surger*” OR Phakectomy OR Capsulorhexis OR Capsulorrhexis).” The search was unrestricted in all databases except Scopus, where it was limited to titles and abstracts. Supplementary Table S1 provides a detailed outline of the search terms used and the results obtained from each database. Furthermore, we manually searched the reference lists of relevant trials to ensure thoroughness and avoid missing pertinent studies.

### Eligibility criteria

RCTs meeting the following Population, Intervention, Control, and Outcome (PICO) criteria were eligible for inclusion: Population (P): Patients undergoing any type of cataract surgery, including phacoemulsification or capsulorhexis. Intervention (I): intracameral moxifloxacin, regardless of the dosing regimen. Control (C): Normal saline intracameral injection or no intracameral antibiotic injection. Outcomes (O): The primary outcome was the incidence of endophthalmitis. Secondary outcomes included changes in endothelial cell count (ECC) and central corneal thickness (CCT).

Studies were excluded based on the following criteria: quasi-randomized design; investigation of combined intracameral antibiotic injections; use of another active antibiotic comparator instead of a placebo or no-treatment control; publication as conference abstracts or proceedings; or study design as observational studies, *in vitro* studies, or reviews.

### Study selection

A total of two reviewers independently screened and selected studies using the Covidence software. After automatically removing duplicates, the remaining unique articles underwent a two-phase screening process. We screened titles and abstracts, then assessed the full text of potentially eligible studies. Discrepancies between the reviewers were resolved through discussion until consensus was reached.

### Data extraction

The data extraction process involved the creation of an Excel spreadsheet, which underwent pilot testing. The extraction form was organized into three main sections: (A) Study characteristics: Study ID, country, study design, total number of patients, treatment protocols, topical antibiotic use, steroid use, surgery type, primary outcome, key inclusion criteria, and follow-up duration; (B) participant baseline characteristics: age and sex; and (C) outcome data: endophthalmitis, changes in ECC, and changes in CCT.

Data were independently extracted by two reviewers (D.A. and R.A.A.). Any discrepancies were resolved through discussion and consultation with a senior author. Event numbers and the total number of participants were extracted for dichotomous data. For continuous data, means and standard deviations were collected. We utilized the formulas proposed by Wan et al. (21) to convert data presented as medians with interquartile ranges or ranges into means and standard deviations.

### Risk of bias and the certainty of the evidence

The methodological quality and the risk of bias for each RCT were assessed using the revised Cochrane Collaboration’s Risk of Bias tool (ROB 2) (22). The two reviewers independently evaluated each study across domains such as selection bias, performance bias, reporting bias, and attrition bias. Disagreements were resolved through consensus. In addition, the overall certainty of the evidence was assessed using the Grading of Recommendations, Assessment, Development, and Evaluation (GRADE) approach (23, 24). This framework considers factors such as risk of bias, inconsistency, indirectness, imprecision, and publication bias. Each factor was carefully assessed, and the rationale for each judgment was documented, with any discrepancies resolved through discussion.

### Statistical analysis

Statistical analyses were conducted using Stata MP version 18 (Stata Corp.). The risk ratio (RR) was computed for dichotomous outcomes, while the mean difference (MD) was used for continuous outcomes, with both presented alongside their corresponding 95% confidence intervals (CIs). The default analysis model was a fixed effects model; however, a random effects model was used if there was significant heterogeneity. Heterogeneity was evaluated using the chi-squared (*χ*^2^) test and the *I*^2^ statistic. A *p*-value less than 0.1 for the *χ*^2^ test or an *I*^2^ value of 50% or higher indicated significant heterogeneity. Assessment of publication bias was not performed because all analyzed outcomes included fewer than 10 RCTs (25).

## Results

### Search results and study selection

**Figure 1:**
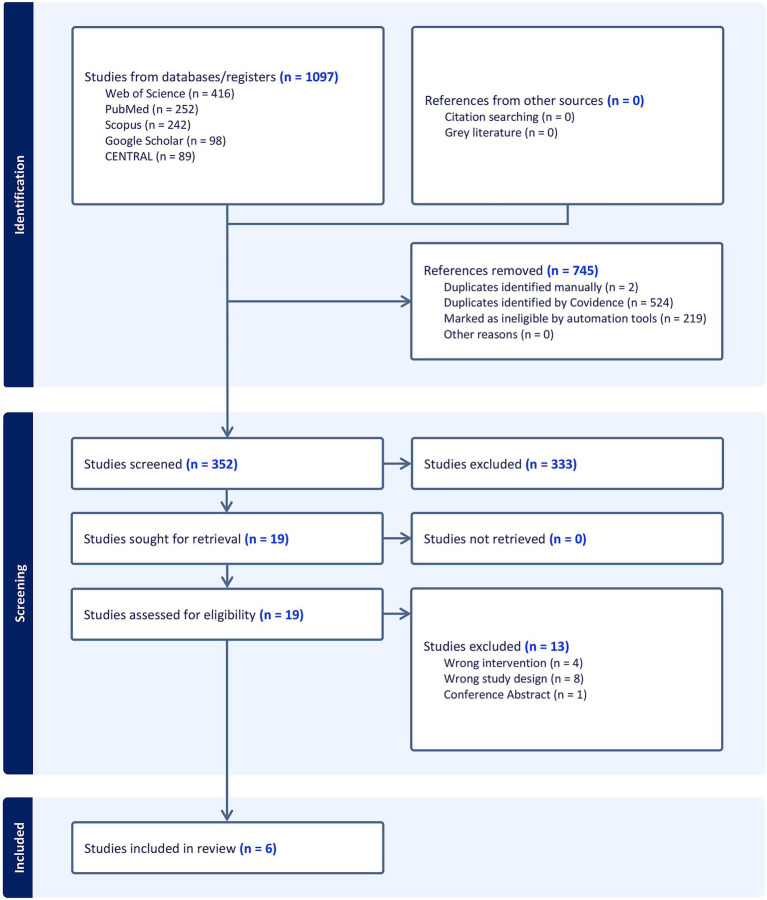
PRISMA flowchart of the screening process.

The initial literature search identified 1,097 records. After automatically removing 745 irrelevant records, the titles and abstracts of the remaining 352 articles were screened. This led to the exclusion of 333 studies that did not meet the inclusion criteria. Consequently, 19 articles underwent full-text assessment for eligibility, of which 13 studies were excluded for various reasons (Supplementary Table S2). Ultimately, six RCTs (11, 14–18) were included in the qualitative and quantitative synthesis (Figure 1).

### Characteristics of the included studies

A total of six RCTs involving 4,438 patients were included in our pooled analysis (11, 14–18). All RCTs investigated intracameral moxifloxacin versus a control (either no injection or balanced salt solution) for prophylaxis after phacoemulsification surgery, using various treatment protocols. The moxifloxacin dose, for instance, ranged from 150 μg to 500 μg per injection. All trials were effectively open-label or had unclear blinding, except for Melega et al., which was a partially masked RCT (18). All included trials administered postoperative topical steroids and antibiotics as adjuvant therapy. The moxifloxacin group included 2,258 eyes, while the control group included 2,190 eyes. Detailed information on study characteristics and patients’ baseline data is provided in Table 1.

**Table 1:** Summary of the characteristics of the included RCTs.

Study ID	Study design	Country	*n*	Mfx concentration and preparation	Ctrl	Topical antibiotic use	Steroid use	Primary outcome	Follow-up duration	Age (Years), Mean (SD)		Sex (Female), %	
										Mfx	Ctrl	Mfx	Ctrl
Amer et al. (2013) (11)	Single-center RCT	Egypt	60 eyes of 60 patients	0.1 mL of 0.5% Mfx (Vigamox)	No intracameral antibiotic injection	Postoperative topical antibiotics were used	Postoperative topical steroids were used	Safety was evaluated by visual rehabilitation, anterior chamber reaction, corneal endothelial cell density, and pachymetry.	24 weeks	65.1 (±7.8)		13 (43.3)	14 (46.7)
Ashraf et al. (2022) (17)	Multi-center RCT	Egypt	60 eyes of 60 patients	0.1 mL (0.5 mg) of an undiluted 0.5% Mfx solution (Vigamox)	No intracameral antibiotic injection	Postoperative moxifloxacin 0.5% eye drops were used for 2 weeks	Postoperative prednisolone acetate 1% eye drops were used for 2 weeks	Safety of prophylactic intracameral moxifloxacin injection.	12 weeks	59.2 (±5.05)	58.77 (±4.43)	19 (63.3)	16 (53.3)
Lane et al. (2008) (14)	Single-center RCT	USA	57 eyes of 47 patients	0.050 mL (250 μg) of an undiluted 0.5% Mfx solution	An equal volume (0.050 mL) of a balanced salt solution	Preoperative moxifloxacin 0.5% eye drops and postoperative moxifloxacin 0.5% eye drops for 7 days were used	Postoperative prednisolone acetate 1% eye drops were used, tapered over 4 weeks	Posterior and anterior segment safety, with macular thickness measured by OCT as the primary evaluator.	12 weeks	74 (±9.3)		28 (59.6)	
Malik et al. (2016) (16)	Single-center RCT	India	60 eyes of 60 patients	0.1 mL of 0.5% Mfx (Occumox)	No intracameral antibiotic injection	Postoperative topical antibiotics were used	Postoperative topical steroids were used	The effect of intracameral moxifloxacin on the morphology and cell density of the corneal endothelium.	6 weeks	64.2 (±7.8)		28 (47.0)	
Melega et al. (2018) (18)	Single-center RCT	Brazil	3,640 eyes of 3,640 patients	0.03 mL (150 μg) of undiluted 0.5% Vigamox	No intracameral antibiotic injection	Postoperative 0.5% moxifloxacin eye drops were used for 7 days. No preoperative antibiotics were used	Postoperative 0.1% dexamethasone eye drops were used, tapered over 4 weeks	The incidence of acute endophthalmitis.	6 weeks	68.50 (±9.72)		892 (49.06)	876 (48.08)
Shakeel et al. (2020) (15)	Single-center RCT	Pakistan	571 eyes of 571 patients	0.1 mL of 0.5% Mfx was injected intracamerally	No intracameral antibiotic injection	Preoperative topical ciprofloxacin and postoperative chloramphenicol eye drops for 4 weeks were used	Intraoperative subconjunctival triamcinolone and postoperative dexamethasone eye drops for 4 weeks were used	Efficacy and safety in preventing postoperative endophthalmitis.	4 weeks	60 (±4.9)		400 (44.0)	

Mfx, Moxifloxacin; RCT, Randomized Controlled Trial; SD, Standard Deviation; USA, United States of America.

### Risk of bias and the certainty of the evidence

**Figure 2:**
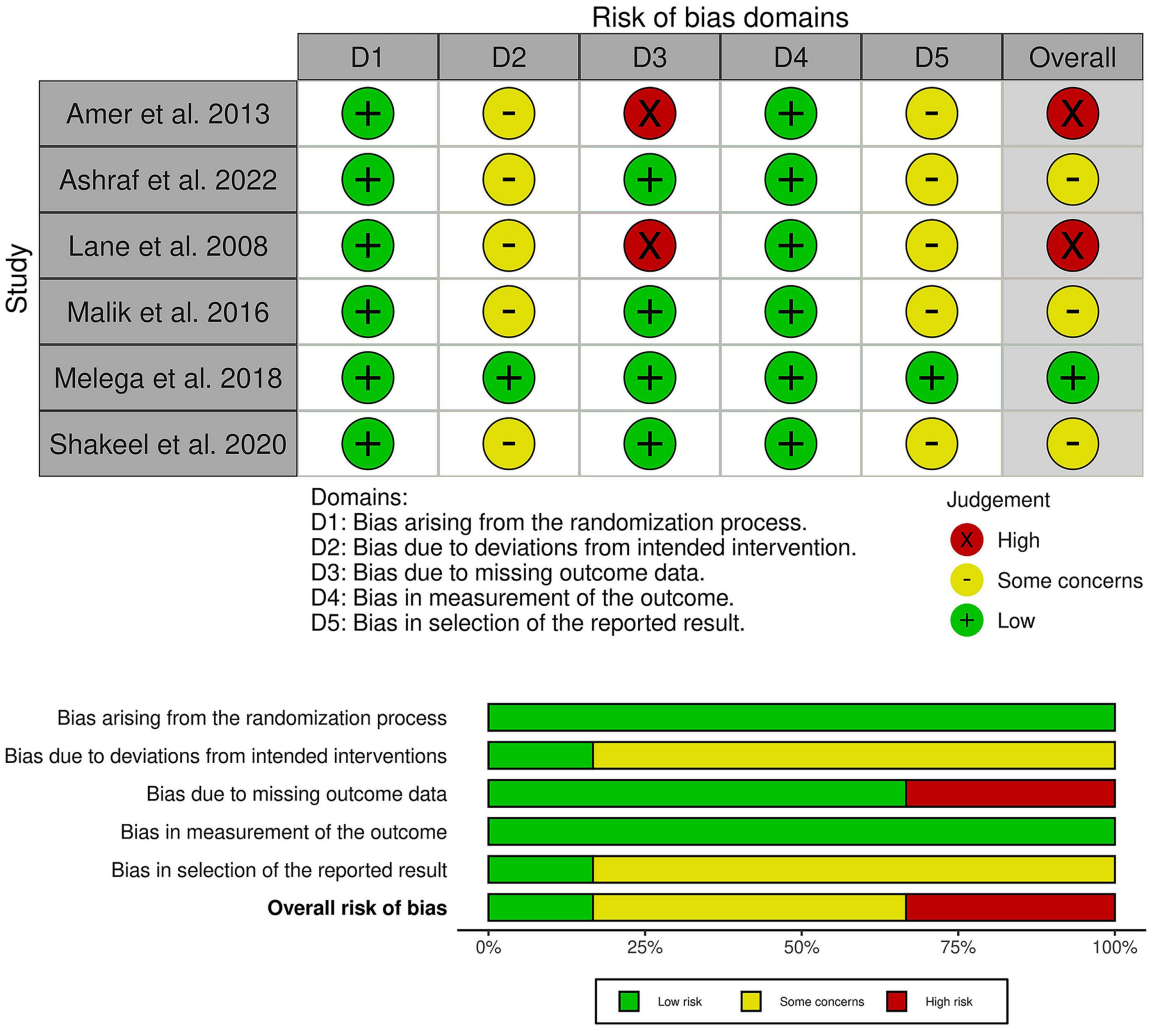
Quality assessment of risk of bias in the included trials. The upper panel presents a schematic representation of the risk of specific types of bias in the individual studies (low = green, unclear = yellow, and high = red). The lower panel presents the overall risk of bias across all included studies for each domain (low = red, unclear = yellow, and high = red).

Among the included trials, one trial showed a low risk of bias (18), three trials showed some concerns (15–17), and two trials had an overall high risk of bias (11, 14) (Figure 2). Significant concerns about missing outcome data, specifically attrition bias, led to the downgrading of the high-risk trials (11, 14). In addition, most other trials raised some concerns due to deviations from the intended intervention and bias in outcome measurement, reflecting the use of unblinded participants and assessors. Finally, selective reporting bias was a common concern due to the lack of pre-registered study protocols. Furthermore, the outcome-based certainty of the evidence is detailed in Table 2.

**Table 2:** Grade evidence profile.

Certainty assessment							Summary of findings				
Participants (studies) Follow-up	Risk of bias	Inconsistency	Indirectness	Imprecision	Publication bias	Overall certainty of the evidence	Study event rates (%)		Relative effect (95% CI)	Anticipated absolute effects	
							With [Control]	With [Moxifloxacin]		Risk with [Control]	Risk difference with [Moxifloxacin]
Endophthalmitis											
4,388 (5 RCTs)	Serious^a^	Not serious	Not serious	Serious^b^	None	⨁⨁◯◯ Low^a,b^	11/2160 (0.5%)	1/2228 (0.0%)	RR 0.22 (0.07 to 0.77)	11/2160 (0.5%)	4 fewer per 1,000 (from 5 fewer to 1 fewer)
Changes in endothelial cell count											
3,750 (3 RCTs)	Not serious	Not serious	Not serious	Very serious^b^	None	⨁⨁◯◯ Low^b^	–	–	–	1882	MD 22.17 higher (from 8.53 lower to 52.88 higher)
Changes in central corneal thickness											
3,750 (3 RCTs)	Not serious	Not serious	Not serious	Serious^b^	None	⨁⨁⨁◯ Moderate	–	–	–	1882	MD 0.03 lower (from 0.36 lower to 0.31 higher)

CI: confidence interval; MD: mean difference; RR: risk ratio; aMost trials showed either some concerns or a high risk of bias. bA wide confidence interval that did not exclude the possibility of appreciable harm/benefit.

### Primary outcome: endophthalmitis

**Figure 3:**
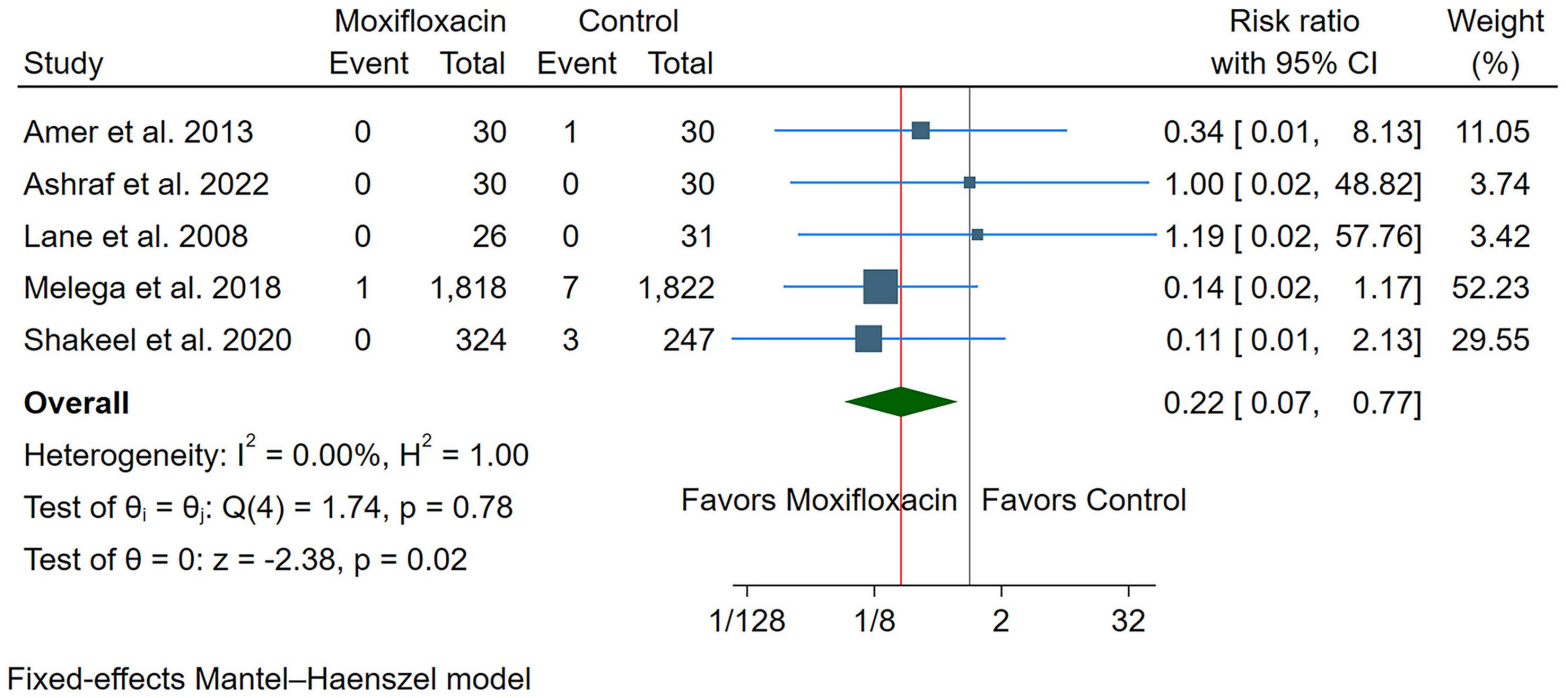
Forest plot of the primary outcome (endophthalmitis). CI: Confidence interval.

Intracameral moxifloxacin significantly reduced the incidence of postoperative endophthalmitis compared to the control (*n* = 5 RCTs, RR: 0.22, 95% CI [0.07, 0.77], *p* = 0.02) (Figure 3). The pooled analysis showed no heterogeneity (*I*^2^ = 0%, *p* = 0.78). A sensitivity analysis excluding studies with a high risk of bias demonstrated that the effect remained statistically significant (*n* = 3 RCTs, RR: 0.183, 95% CI 0.04, 0.87, *p* = 0.03), with no evidence of heterogeneity (*I*^2^ = 0%, *p* = 0.65). In dose-based subgroup analyses of the primary outcome, neither the ≥500 μg subgroup (3 RCTs; RR 0.18, 95% CI 0.02–1.59; *p* = 0.12) nor the <500 μg subgroup (2 RCTs; RR 0.14, 95% CI 0.02–1.16; *p* = 0.07) achieved statistical significance. Nonetheless, both subgroups consistently trended toward reduced rates of endophthalmitis, with no observed heterogeneity (*I*^2^ = 0%), and no evidence of differential effects by dose was detected within the available data (Supplementary Table S3).

### Secondary outcomes

**Figure 4:**
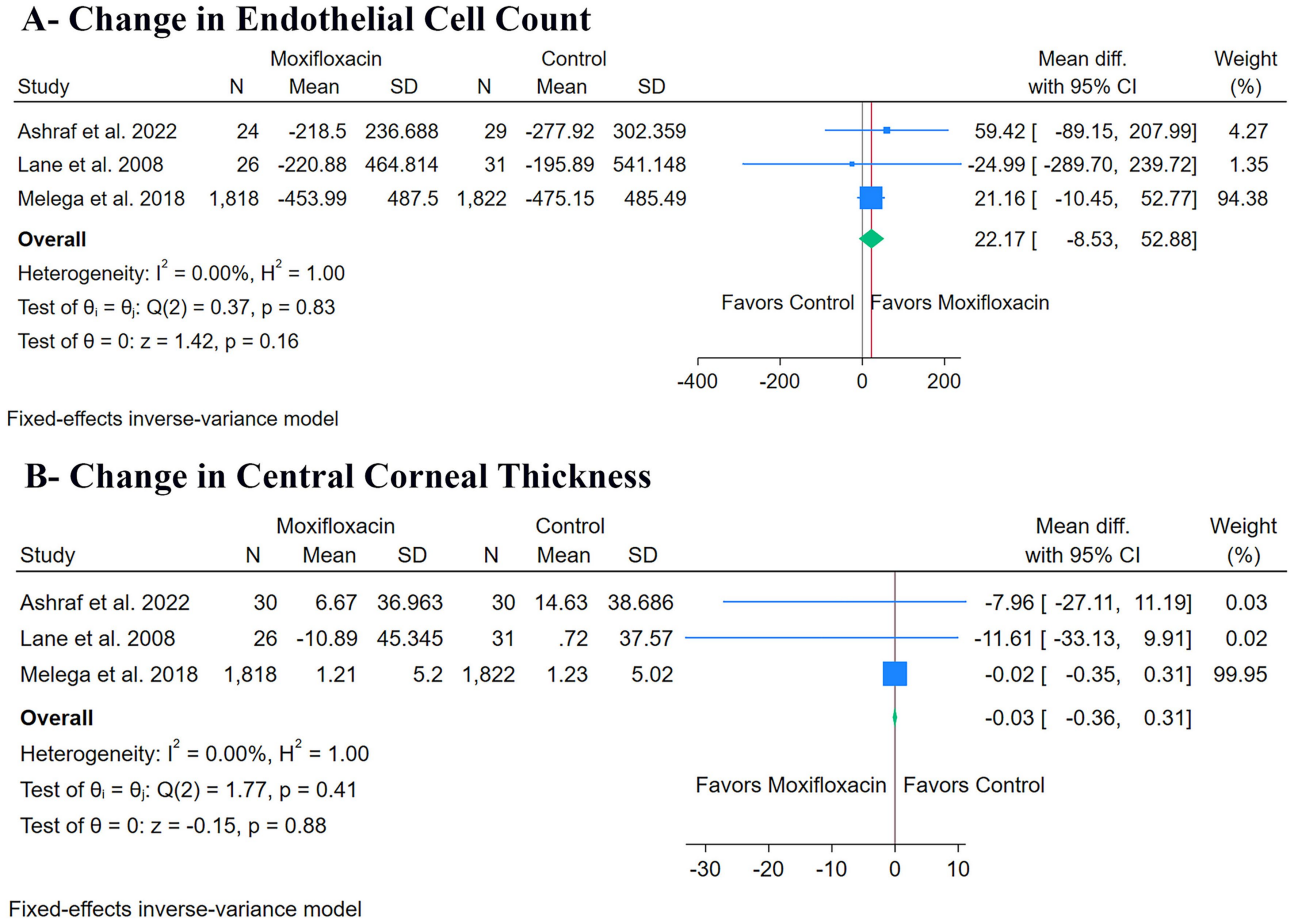
Forest plots of the secondary outcomes: **(A)** change in endothelial cell count, and **(B)** change in central corneal thickness.

There was no significant difference between the moxifloxacin and control groups regarding changes in ECC (*n* = 3 RCTs, MD: 22.17, 95% CI [−8.53, 52.88], *p* = 0.16) (Figure 4A) or changes in CCT (n = 3 RCTs, MD: -0.03, 95% CI [−0.36, 0.31], *p* = 0.88) (Figure 4B). The pooled studies were homogeneous for both changes in ECC (*I*^2^ = 0%, *p* = 0.83) and changes in CCT (*I*^2^ = 0%, *p* = 0.41).

## Discussion

After pooling data from six RCTs involving over 4,400 patients, intracameral moxifloxacin was associated with a 78% relative risk reduction in postoperative endophthalmitis. This major protective effect occurred without any noticeable negative impact on the corneal endothelium, as there were no significant differences in postoperative changes in ECC or CCT compared to the control groups. The groundbreaking ESCRS multi-center study provided the foundation for intracameral antibiotic prophylaxis, as it was the first large study to demonstrate the benefits of this approach (6, 26). The ESCRS trial showed that intracameral cefuroxime reduced the risk of endophthalmitis by nearly fivefold, and subsequent meta-analyses have confirmed these findings, establishing intracameral prophylaxis as standard practice (27–29).

Notable retrospective studies have reported a substantial decrease in endophthalmitis rates following the implementation of intracameral moxifloxacin (13, 30, 31). The present meta-analysis strengthens the evidence base by moving from observational data to Level 1 evidence through pooled RCTs, thereby validating moxifloxacin’s effectiveness with greater internal validity. In addition, moxifloxacin offers potential pharmacological advantages compared to cefuroxime, such as broader activity against Gram-negative organisms and availability as a prepared, preservative-free, isotonic solution, which eliminates the risks associated with off-label preparations (10, 32, 33).

Moreover, this meta-analysis confirmed intracameral moxifloxacin’s safety, particularly concerning the corneal endothelium. The pooled analysis found no statistically significant difference between the moxifloxacin and control groups regarding postoperative changes in ECC or CCT. Accordingly, our result offers robust evidence, synthesized from RCTs, suggesting that moxifloxacin, at the tested doses, does not cause clinically significant endothelial toxicity beyond the expected surgical trauma. The safety outcomes reported in the included individual RCTs were consistent with this finding (14, 17, 18). Although the pooled ECC and CCT analyses did not demonstrate significant differences between the groups, the confidence intervals, especially for ECC, were relatively wide, reflecting the smaller sample sizes available for these outcomes. In clinical practice, endothelial cell loss of approximately 10% or more, or a postoperative increase in CCT exceeding 30–40 μm, is typically regarded as clinically meaningful following routine phacoemulsification (34, 35). Importantly, the changes reported in the included RCTs (14, 17, 18) remained well below these thresholds, supporting the absence of any clinically significant endothelial damage, despite the statistical imprecision.

Although not meeting the inclusion criteria for this meta-analysis, additional studies have assessed the safety of intracameral moxifloxacin and found comparable results. Espiritu et al. and Arbisser et al. reported no adverse effects on the endothelium, anterior chamber inflammation, or macular changes linked to the antibiotic (36, 37). Preclinical and animal model studies also support these clinical observations, demonstrating that moxifloxacin is non-toxic to sensitive intraocular tissues, with no significant histologic or functional damage to the corneal endothelium or retina following intraocular administration (38, 39). The consistent safety signals observed across all levels of evidence, from basic science to pooled RCT data, strongly support moxifloxacin’s corneal safety.

Antimicrobial resistance is an increasingly important consideration when interpreting the benefits of intracameral fluoroquinolones. Surveillance studies have documented decreasing *in vitro* susceptibility and rising resistance to fourth-generation fluoroquinolones, including moxifloxacin, among staphylococcal isolates from postoperative endophthalmitis and ocular surface infections, underscoring the need for prudent use of these agents (40). Likewise, recent systematic research suggests evolving resistance patterns among ocular pathogens and calls for explicit antimicrobial stewardship strategies in ophthalmology (41). Antiseptics assume a central role because they show broad-spectrum, non-selective antimicrobial activity and do not select for specific resistance mechanisms. Povidone–iodine remains the cornerstone of preoperative antisepsis in cataract surgery, with robust evidence demonstrating a substantial reduction in conjunctival bacterial load and endophthalmitis risk (42). More recently, novel ophthalmic antiseptic formulations, such as hypochlorous acid-based solutions or polymeric complexes, have demonstrated broad antibacterial and antiviral activity *in vitro*, supporting their use as adjuncts or alternatives to topical antibiotics in perioperative care (43). Beyond intracameral prophylaxis, the use of postoperative topical antibiotics also warrants particular attention from an antimicrobial stewardship perspective. Postoperative topical antibiotics require particular attention from an antimicrobial stewardship perspective because they expose the ocular surface flora to repeated, subtherapeutic drug concentrations over several days, creating stronger and more sustained selective pressure than a single intracameral dose administered at the end of surgery. Repeated topical fluoroquinolone exposure has been associated with increased resistance among ocular surface isolates in clinical settings (44), and reviews of cataract prophylaxis emphasize that topical antibiotic practices can shape resistance trends and should be used judiciously (40, 45). In parallel, evidence syntheses indicate that intracameral antibiotics provide the most consistent reduction in endophthalmitis risk, whereas topical antibiotics alone have not shown a consistent protective effect (29), supporting an antimicrobial stewardship approach that prioritizes proven measures (e.g., povidone–iodine antisepsis) and critically reevaluates routine postoperative topical antibiotic courses, especially when intracameral prophylaxis has already been used (46).

In clinical practice, intracameral moxifloxacin is often combined with postoperative topical fluoroquinolones. However, this combination may represent redundant antimicrobial exposure, as intracameral administration achieves high intraocular concentrations at the time of greatest contamination risk. The incremental benefit of adding postoperative topical moxifloxacin in this context remains uncertain, whereas prolonged topical exposure may increase selective pressure on the ocular surface flora. From an antimicrobial stewardship perspective, routine dual use should be critically vevaluated, particularly when robust antiseptic measures are employed, such as povidone–iodine, which remains the only intervention consistently shown to reduce endophthalmitis risk across settings (29, 46).

This review’s main methodological advantage is that it is the first meta-analysis to synthesize only Level 1 evidence from RCTs when evaluating intracameral moxifloxacin. This results in stronger internal validity compared to previous reviews using observational data (27–29). Despite this significant strength, some limitations should be acknowledged. First, two of the six trials were judged to have a high risk of bias, while the remaining studies raised some concerns. The potential impact on the certainty of the evidence was substantially mitigated by the meta-analysis’s statistical design, which used a weighted average for the final pooled estimate and the inclusion of the large, low-bias study by Melega et al. (18), contributing the majority of the statistical information and thereby minimizing the impact of the smaller, higher-risk trials. In addition, a sensitivity analysis excluding the two high-risk trials demonstrated that the protective effect remained statistically significant and clinically meaningful (*n* = 3 RCTs, RR: 0.183, 95% CI 0.04, 0.87, *p* = 0.03), with no heterogeneity, further supporting the robustness of the primary finding. Second, although statistical heterogeneity was low (*I*^2^ = 0%), meaningful clinical heterogeneity existed across the trials. This included variability in intracameral moxifloxacin dosing (150–500 μg), differences in the concomitant use of topical antibiotics, and heterogeneity in control interventions (balanced salt solution versus no intracameral injection). Such clinical variability may influence treatment effects and safety profiles and is not necessarily captured by quantitative heterogeneity metrics. Therefore, statistical homogeneity should not be interpreted as equivalence in the clinical context, and the pooled estimates should be applied with appropriate clinical judgment. Importantly, because all included trials administered postoperative topical antibiotics, the independent effect of intracameral moxifloxacin cannot be fully isolated, and the observed benefit likely reflects its use as part of a combined prophylactic strategy rather than as a standalone intervention. Third, the safety outcome analysis (ECC and CCT) used a smaller patient subset, possibly reducing the statistical power to identify minor differences. Finally, due to the limited number of studies included (fewer than 10), a formal assessment of publication bias was not conducted, and its potential impact cannot be completely dismissed.

## Conclusion

Prophylactic intracameral moxifloxacin significantly reduces the incidence of postoperative endophthalmitis after cataract surgery. This considerable benefit was observed without detectable adverse effects on corneal endothelial parameters, as no statistically significant differences in ECC or CCT were found, although safety conclusions are limited by the small subset of patients evaluated. Dose-based subgroup analyses of the primary outcome showed no statistically significant differences between higher (≥500 μg) and lower (<500 μg) doses, suggesting no clear dose–response effect within the available evidence.

## Data Availability

The original contributions presented in the study are included in the article/Supplementary material, further inquiries can be directed to the corresponding author/s.
