# Supplementary material for: Intracameral moxifloxacin for endophthalmitis prophylaxis after cataract surgery: a systematic review and meta-analysis
Source: Front Med (Lausanne). 2026 Jan 8;12:1704056. doi: 10.3389/fmed.2025.1704056 (PMC12823917; doi:10.3389/fmed.2025.1704056)
Supplement: Supplementary file 1 [file Table_1.docx]

**Table S1**. Search Strategy.

| **Database** | **Search Terms** | **Search Field** | **Search Results** |
| --- | --- | --- | --- |
| PubMed | (Moxifloxacin OR Avelox OR Moxeza OR Vigamox OR Actira OR Izilox OR Octegra OR Proflox) AND (“Cataract Extract*” OR Phacoemulsification OR “Cataract Surger*” OR Phakectomy OR Capsulorhexis OR Capsulorrhexis) | All Fields | 252 |
| Cochrane | (Moxifloxacin OR Avelox OR Moxeza OR Vigamox OR Actira OR Izilox OR Octegra OR Proflox) AND (“Cataract Extract*” OR Phacoemulsification OR “Cataract Surger*” OR Phakectomy OR Capsulorhexis OR Capsulorrhexis) | All Text | 89 |
| Web of Science | (Moxifloxacin OR Avelox OR Moxeza OR Vigamox OR Actira OR Izilox OR Octegra OR Proflox) AND (“Cataract Extract*” OR Phacoemulsification OR “Cataract Surger*” OR Phakectomy OR Capsulorhexis OR Capsulorrhexis) | All Fields | 416 |
| Scopus | TITLE-ABS ( ( Moxifloxacin OR Avelox OR Moxeza OR Vigamox OR Actira OR Izilox OR Octegra OR Proflox ) AND ( "Cataract Extract*" OR Phacoemulsification OR "Cataract Surger*" OR Phakectomy OR Capsulorhexis OR Capsulorrhexis ) ) | Title, Abstracts | 242 |
| Google Scholar | (Moxifloxacin OR Avelox OR Moxeza OR Vigamox OR Actira OR Izilox OR Octegra OR Proflox) AND (“Cataract Extract*” OR Phacoemulsification OR “Cataract Surger*” OR Phakectomy OR Capsulorhexis OR Capsulorrhexis) | All Fields | 98 |

**Table S2**. Excluded records in full-text screening.

| **Title** | **Published Year** | **Exclusion reason** |
| --- | --- | --- |
| A randomized, prospective, observer-masked study comparing dropless treatment regimen using intracanalicular dexamethasone insert, intracameral ketorolac, and intracameral moxifloxacin versus conventional topical therapy to control postoperative pain and | 2023 | Wrong intervention |
| Efficacy of injecting intra-vitreal moxifloxacin in acute post-operative endophthalmitis. | 2023 | Wrong study design |
| Clinical features and microbiology of post-cataract surgery endophthalmitis with and without intracameral moxifloxacin prophylaxis: Endophthalmitis prophylaxis study report 3. | 2022 | Wrong study design |
| Long-term safety of intracameral moxifloxacin after cataract surgery. | 2017 | Wrong study design |
| Aqueous humor penetration and biological activity of moxifloxacin 0.5% ophthalmic solution alone or with dexamethasone 0.1. | 2017 | Wrong intervention |
| Safety of prophylactic intracameral moxifloxacin use in cataract surgery. | 2012 | Wrong study design |
| Comparison of 2 moxifloxacin regimens for preoperative prophylaxis: prospective randomized triple-masked trial. Part 2: residual conjunctival flora. | 2008 | Wrong study design |
| Safety of irrigating solution containing moxifloxacin in cataract surgery | 2015 | Wrong study design |
| Safety and efficacy of intracameral injection of dexamethasone and moxifloxacin at the end of cataract surgery | 2022 | Wrong intervention |
| Effect of intracameral moxifloxacin on endothelial cell count and endophthalmitis prophylaxis after cataract surgery: effect of intracameral moxifloxacin | 2024 | Wrong study design |
| Efficacy of Intracameral moxifloxacin in Cataract surgery to prevent post-operative Endophthalmitis | 2010 | Wrong study design |
| Antibacterial Drugs in the Prevention of Complications of Cataract Phacoemulsification | 2023 | Wrong intervention |
| Evaluation and comparison of macular and choroidal thickness after intracameral moxifloxacin for prevention of postcataract endophthalmitis | 2018 | Conference Abstract |

**Table S3**: Subgroup analysis for the primary outcome.

| Outcome | Subgroup | Number of studies | Risk ratio | 95% CI | Heterogeneity (I^2^) | p value |
| --- | --- | --- | --- | --- | --- | --- |
| Endophthalmitis rate | ≥500 µg | 3 | 0.18 | 0.02, 1.59 | 0% | 0.12 |
|  | <500 µg | 2 | 0.14 | 0.02, 1.16 | 0% | 0.07 |
